# Macrophage Polarization: Decisions That Affect Health

**DOI:** 10.4172/2155-9899.1000364

**Published:** 2015-10-30

**Authors:** Charles D Mills, Robert A Harris, Klaus Ley

**Affiliations:** 1 BioMedical Consultants, Marine on St. Croix, MN 55047 USA; 2 Applied Immunology & Immunotherapy, Department of Clinical Neuroscience, Karolinska Institutet, CMM, Karolinska Hospital, Stockholm, Sweden; 3 La Jolla Institute for Allergy and Immunology, La Jolla, CA 92037 USA

**Keywords:** Macrophage, Innate immunity, Polarization M1, M2, iNOS, Arginase, Th1, Th2

## Commentary

The purpose herein is to introduce the second part of the series on macrophage ‘polarization’ by illuminating some basic properties of the immune system’s most important and uniquely multi-talented leukocyte.

Macrophages were the first separate leukocytes to appear in evolution about 900 million years ago [1].

They are the most abundant leukocytes in all animals.

Macrophages are located in virtually all tissues: they are the tissue ‘sentinels’ [2,3].

Macrophages possess unique ‘plasticity’ [4,5] that endows them with the ability to repair or to kill: exhibit polar-opposite functions [4,6–10]. Both types of function are necessary for the survival of animals.

### Repair – *constructive* activity

Required to help repair and replace cells/tissues lost to senescence or damage.

1. By elimination through engulfment and digestion
2. Through the production of growth and repair molecules
3. By helping in the production of extracellular matrices for intercellular support

### Kill – *destructive* activity

Provides primary host protection against pathogens in all animals

1. By direct killing activity through phagocytosis and the production of reactive oxygen and nitrogen species [1,3,8]
2. By instructing other innate leukocytes (e.g., neutrophils) to aid in pathogen elimination [11]
3. Through antigen presentation to T and B cells resulting in more specific and effective defenses against pathogens and altered self [12,13]

The constructive – repair – activity is commonly called M2 and the destructive – kill – activity of macrophages is called M1 [10,14]. M2- and M1-type activities occur throughout the animal kingdom and are normally induced by macrophages sampling their environs for Damage- or Pathogen- Associated Molecular Patterns (DAMPs and PAMPs) [15]. By sensing whether to exhibit constructive or destructive activities macrophages are uniquely able to protect hosts in ways best suited to correcting varying non-infectious or infectious threats to hosts.

Macrophages were renamed M2/Heal and M1/Inhibit [14] in part because these repair or kill activities are associated with the production of Ornithine or Nitric Oxide, respectively, and other growth-promoting or growth –inhibiting molecules [4,10]. Also, importantly, these very different innate protective activities do not require T cells/ adaptive immunity, though macrophages can undergo further “activation” [16] or ‘alternative activation’ [17] by antigen-specific T cells/adaptive immunity [10]. In this regard, M2/Heal and M1/Inhibit-type activities precede the appearance of T cells/adaptive immunity in evolution by about 500 million years [1,15]. In fact, the ability of cells to polarize functions is an evolutionarily ancient property even exhibited by single-celled animals such as amoeba [1,18]. As mentioned, macrophages were the first leukocyte to evolve that specializes in protecting other cells [1].

It is useful to think of macrophage polarization as ‘decision-making’ that results in distinct cellular functions that affect host health in very different ways. Macrophage decision-making results from their versatile *Sample* function that activates M2/*Heal* (Repair) or M1/*Inhibit* (Kill)-type functions that in turn can result in their *Present* function that is necessary to activate lymphocytes. Together these basic macrophage activities can be summarized as SHIP functions (*Sample, Heal, Inhibit* and *Present* [antigen]) [19,20].

Upon encounters with M1- or M2-type macrophage activities other leukocytes are also caused to make decisions – to polarize. For example, Th1- or Th2-type responses, which are characterized by the preferential production of the cytokines IFN-γ or IL-4, respectively [21–23]. These different cytokines in turn stimulate cytotoxic NK and T cells, or B cells/antibody production, respectively. Finally, Th1- or Th2-type polarized T cell responses can further amplify M1/Inhibit or M2/Heal-type activities [19]. Macrophages also cause other immune-related decisions to be made including complement activation and coagulation, but are beyond the scope of this commentary [24].

Thus the necessary epicenter of immune systems is macrophages that make decisions resulting in functions that directly or indirectly affect host health in profoundly different ways – literally life or death decisions [4]. This is the essence of macrophage polarization.

**Figure 1:**
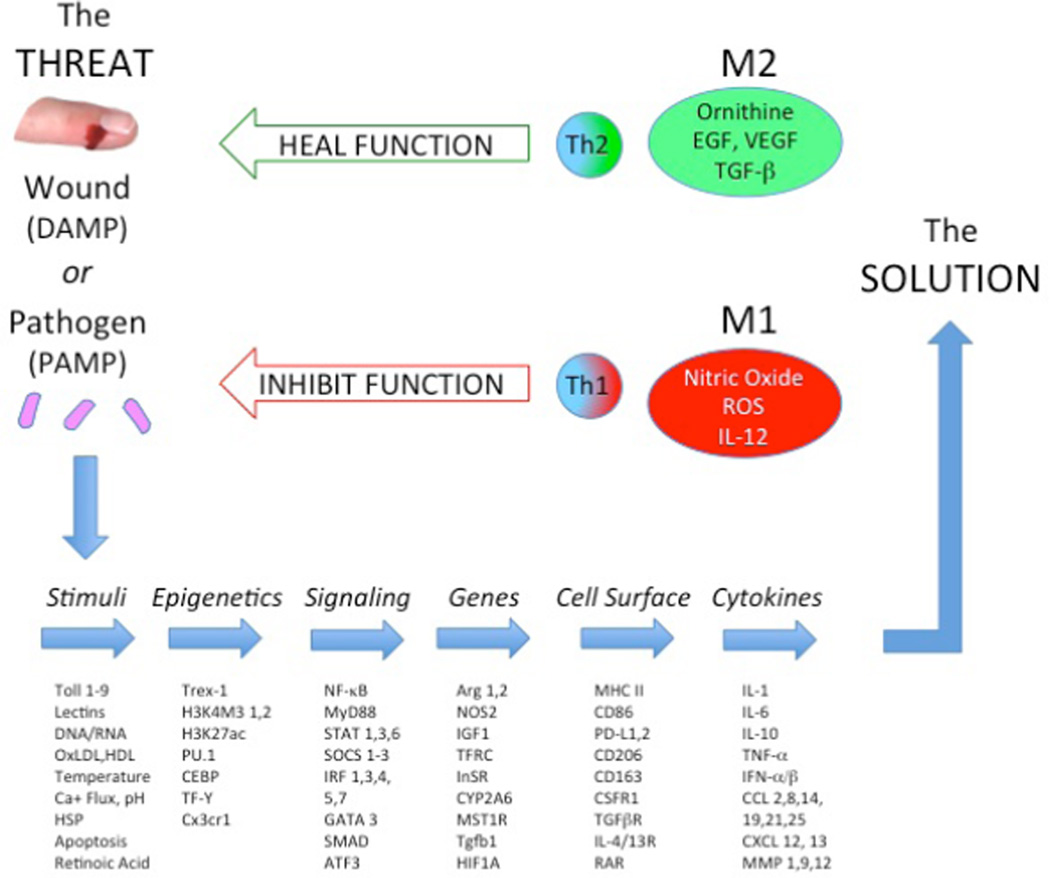
Macrophages encounter many different types of ‘Threats’ (i.e., Stimuli) that result in changes in thousands of molecules as macrophages modulate their physiology to come up with a ‘Solution’ such as M2/*Heal* or M1/*Inhibit*.

Some researchers have posited that there are different types of macrophages (e.g., M2 a/b/c, Type II, regulatory, M4 and Mox macrophages) [25–29]. Others have gone further envisioning macrophages as of part of a continuum [30,31]. But such views arise mainly from changing the rules for characterizing macrophages from their functional activity (like the original definition of macrophage activation meaning killing pathogens) [16] to characterizing them by ‘phenotypes’. The unique ability of macrophages to respond to different types of agonists (stimuli) and to exhibit very different functions (like repair or kill) is accompanied by changes in thousands of different genes, transcription factors, cell surface markers and cytokines, as illustrated in Figure 1. Phenotype means traits or types. Recent transcriptomic or genomic analyses of macrophages has further expanded the list of molecules that change [31], and has importantly illuminated how the uniquely changeable metabolic machinery of macrophages operates. However, changes in molecules such as NF-κB, GATA 3, or HIF are phenotypic ‘traits’ that alone are not sufficient to characterize functional ‘types’ of macrophages. The clinically relevant types of macrophages are those that influence health. In particular, as illustrated in Figure 1, macrophages through their Sample functions, are able to determine the nature of the *Threat* and make decisions that result in *Solutions* through distinct and very different functions like M2/Heal or M1/Inhibit. Without what have come to be called polarized macrophage functions diseases are exacerbated or hosts perish.

Compounding the difficulty in properly assessing macrophage populations is the fact that inflammation is constantly evolving as diseases progress or are eliminated. Furthermore, macrophage functions can vary enormously within different inflammatory microenvironments [32]. For example, during Tuberculosis infections sections of lungs where scarring is ongoing are populated by macrophages with M2/Heal activities, while M1/Inhibit activity is evident in areas where mycobacteria are being killed (33). Assaying macrophages at different times or grinding up whole organs will therefore necessarily reveal mixtures of different macrophages. It is useful to add here that the use of the terms *anti-inflammatory* or *pro-inflammatory* to describe M2/Heal or M1/Inhibit-type macrophage activities has also created some confusion. Either type of response causes inflammation, for example, as anyone who has had a healing wound knows. Not unlike the inadequacy of phenotyping macrophages mentioned above, it is therefore more clinically relevant to describe what type of macrophage functions (like M2/Heal or M1/Inhibit) are present than to use the broad term inflammation.

To try and address the types of confusion that has arisen from assessing macrophages by phenotypes instead of biological functions, and at different times, in different microenvironments and in different species, there was a laudable recent attempt in *Immunity* to standardize definitions of macrophage populations [34]. A new nomenclature was suggested mainly based on what stimuli were added to macrophages *in vitro*, such as M(IFN-γ) or M(IL-4). However, this nomenclature does not reflect *in vivo* circumstances. The normal primary initiating stimuli for macrophages are not T cell cytokines, like IFN-γ or IL-4, but DAMPs or PAMPs as mentioned earlier. In addition, host genetic factors strongly influence the propensity to polarize to M1 or M2-type functions [10,14]. Following stimulation macrophages direct other innate and adaptive leukocytes in varying ways, and which then also can further amplify or inhibit M2- or M1-type functions. Thus while trying to assign macrophages names based on what T cell-derived (or other) agonist was used is technically useful *in vitro* such a nomenclature incompletely describes how immune responses occur *in vivo*; in fact it is backward. In addition, such a nomenclature does not assess macrophages by their most important characteristic mentioned earlier – their health-impacting functions.

In conclusion, macrophages are the most important leukocytes because of their unique ability to make critical decisions about what functions to manifest, whether insuring tissue integrity or combatting pathogens or altered self. That macrophage populations are complex mixtures of different cells performing varying functions at different times and in different inflammatory microenvironments is not only not surprising, it reflects why macrophages are the most important leukocytes – the ‘Chicken and the Egg’ of immunity [19]. They are able to make the decisions that initiate, prosecute, and conclude inflammation to ensure host health. They are able to polarize. Improper balances of different macrophage functions can of course contribute to (or cause) important diseases, including infections, cancer, autoimmunity and atherosclerosis [10,35,36]. The articles in the first part of this series on ‘macrophage polarization’, and in this second part, help us understand how imbalances in macrophage functions can undermine health, and how one might go about correcting the imbalances through immunomodulation, drugs or other means.
